# Anesthesiology Residency Matching: Key Differentiators in a Post-Step 1 Pass/Fail Era

**DOI:** 10.7759/cureus.108380

**Published:** 2026-05-06

**Authors:** Joshua Kim, Giselle Tran, Kishore Bharadwaj, Annalia Ferrer, Christian Bohringer, Michael S Wong, Hong Liu

**Affiliations:** 1 Anesthesiology, California Northstate University College of Medicine, Elk Grove, USA; 2 Anesthesiology, University of California, Davis, Davis, USA; 3 Surgery/Plastic Surgery, California Northstate University College of Medicine, Elk Grove, USA; 4 Anesthesiology and Pain Medicine, University of California, Davis Health, Sacramento, USA

**Keywords:** academic competitiveness, acgme-accredited programs, anesthesiology residency, graduate medical education, national residency matching program, research experience, residency match

## Abstract

Introduction: In 2022, the USMLE Step 1 transitioned to pass/fail grading, marking a pivotal change in how residency candidates are evaluated. Taking this change into consideration, a survey sent to anesthesiology program directors (PDs) revealed how they frequently ranked letters of recommendation, USMLE Step 2 scores, and letters of recommendation in the top 10 factors for deciding potential candidates to interview. This emphasized how the Step 1 shift prompted PDs to place greater emphasis on other application components. While this change affected all specialties, this study focuses on its impact on the competitiveness of anesthesiology residency applicants.

Method: Using National Residency Matching Program (NRMP) data from 2008 to 2023, matched anesthesiology applicants (MAAs) were compared against unmatched anesthesiology applicants (UAAs) and all matched applicants (AMAs) across key academic (e.g., USMLE Step 1 and Step 2 scores, AOA status, top 40 NIH-funded medical school), experiential (e.g., research experiences, abstracts/presentations/publications, graduate/PhD degrees), and other match-related metrics (e.g., contiguous ranks and number of distinct specialties ranked).

Results: Significant differences were observed among factors involving matched versus unmatched applicants, with Step 2 scores (*p*=1.01 x 10^-6^), research experiences (5.66 x 10^-4^), and publication volume (5.63 x 10^-3^) being primary differentiators for MAA. In contrast, PhD attainment (2.7 x 10^-1^) was not a consistent predictor of match success. Notably, PGY-1 match rates for MD seniors have improved, with 1 in 1.5 applicants matching. On the other hand, PGY-2 match ratios have worsened, with the ratio of total applicants per matched applicant increasing by over 300%. This reflects increased match competitiveness driven by reduced PGY-2 opportunities.

Conclusion: These findings highlight the growing importance of Step 2 scores and research productivity for a successful match into an anesthesiology residency. As the evaluation process continues to evolve, this underscores the need for prospective applicants to prioritize well-roundedness to remain competitive in an increasingly selective residency landscape.

## Introduction

The anesthesiologist shortage in the United States is a multifaceted issue driven by demographic shifts, increasing surgical demand, insufficient graduate medical education funding for residency positions, and workforce dynamics. A growing elderly population, particularly those aged 65 and older, is a major driver of healthcare utilization, with the proportion of surgical services required by this group expected to rise from 31% to 39% by 2034 [1]. The aged population requires a disproportionate number of surgeries and procedures and often has multiple medical comorbidities, such as obesity, diabetes, cardiovascular issues, and chronic pain, which increases the risk for perioperative complications. As a result, both the number of people who need anesthesia care and the level of advanced knowledge required to provide care safely and effectively are expected to rise, putting even more pressure on the current healthcare system [2]. These patterns of disease burden will continue to fuel the demand for surgical and interventional procedures, critical care, and pain management [3]. At the same time, the rise in ambulatory surgery centers and non-operating theater procedures, such as interventional radiology and endoscopy, has expanded the scope of anesthesia services, with major teaching hospitals experiencing an appreciable 13% rise in outpatient surgeries between 2013 and 2019 [4,5]. However, the capacity to meet these demands is constrained by inadequate Graduate Medical Education (GME) funding for new residency positions, which has been frozen since 1997 under the Balanced Budget Act. While efforts like the Resident Physician Shortage Reduction Act of 2021 aim to increase residency slots by 2,000 each year across all specialties between 2023 and 2029, these fall significantly short of addressing the projected shortfall [6].

Meanwhile, the anesthesiology workforce is aging, with 45% of current anesthesiologists older than 55 years of age [6]. Although CRNAs will help alleviate the demand for anesthesia services, physician anesthesiologists receive vastly more clinical training than their counterparts and thus remain integral to cases involving high complexity and patient acuity, particularly in major hospitals in large urban settings where a physician supervisory model remains prevalent [6,7]. While the supply of anesthesiologists has increased steadily and is at an all-time high, this growth is limited by plateaued training output. Understanding the trends in applicant supply, training capacity, and workforce needs is crucial for strategically addressing the systemic factors that will shape the future of anesthesiology as a profession. Each year, candidates from allopathic medical schools apply to anesthesia residency programs all around the country. While some residency PDs prioritize letter of recommendation and clerkship grades as critical forms of evaluation, others place more emphasis on STEP 2 scores along with research publications and experience [8]. Despite the increase in the number of applicants, there is a disproportionate growth when comparing this trend to the number of postgraduate year 1 (PGY-1) and postgraduate year 2 (PGY-2) positions available over time [9]. In this paper, we delve further into the shifting number of PGY-1 and PGY-2 positions and the impact the trend has on the match outcomes of MD seniors in the U.S. In addition, we explore a diverse set of variables involved with the match outcomes for anesthesiology to understand what makes a candidate competitive.

## Materials and methods

The National Residency Matching Program (NRMP) has consistently reported aggregate data regarding matching outcomes by periodically releasing two documents to the public. The first is the Annual Main Residency Match Results and Data [10], which, among other information, has data covering anesthesiology match rates. The second is Charting Outcomes [11], which maps data regarding Step 1 and 2 scores, research experiences, publications, presentations, membership in medical honor societies, graduate education, and match rank behaviors across applicants. Data is not only aggregated across applicants in general, but can be further evaluated for those who successfully matched into anesthesiology and those who were unable to match into anesthesiology, along with information on those who successfully matched into any specialty of their choice. To better characterize the relative competitiveness of matching into anesthesiology, one-way ANOVA tests were performed for each metric provided by the NRMP from 2008 to 2023 to compare matched anesthesiology applicants (MAA), unmatched anesthesiology applicants (UAA), and all matched applicants (AMA). The exception was for mean contiguous ranks, which were likely to be nonparametric, and thus a Kruskal-Wallis rank sum test was used. Subsequently, for statistically significant metrics, t-tests were performed to compare MAAs against UAAs and AMAs, with Bonferroni corrections applied to account for potentially elevated error rates. Once again, for mean contiguous ranks, a nonparametric Wilcoxon signed-rank exact test was used.

## Results

Trends in anesthesiology residency positions

**Figure 1:**
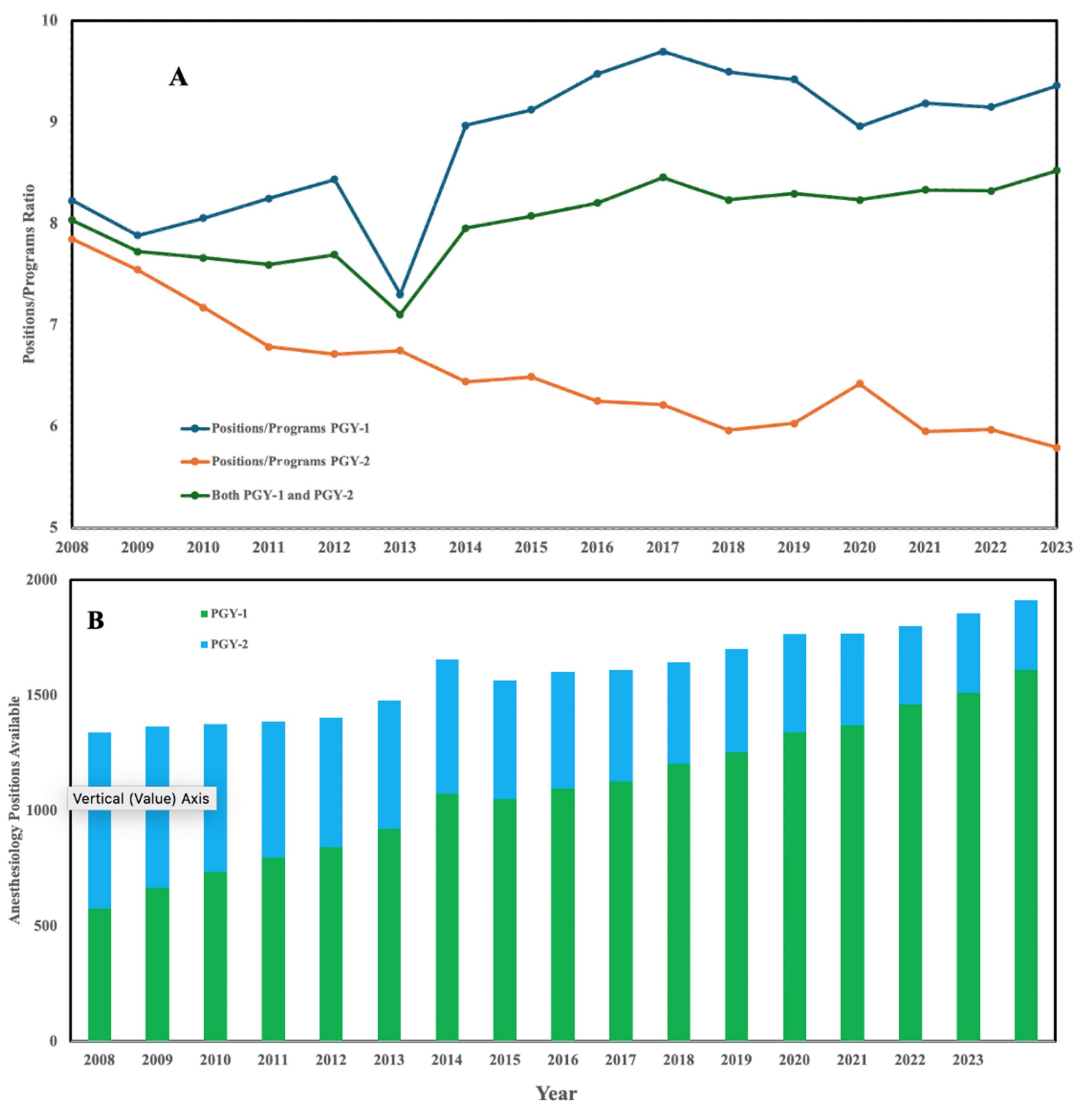
Trends in anesthesiology residency positions Figure 1. Trends in anesthesiology residency positions. A: The PGY-1 positions have nearly doubled, compensating for the decline in PGY-2 positions over time, B: while the overall number of positions remains stable.

NRMP data showcasing the number of anesthesiology residency positions available compared to the number of programs shows a dichotomous trend. The overall availability of positions across programs seems to stay relatively stagnant. However, this stability is largely driven by the rise of average positions per program from PGY-1 programs, which was necessary to compensate for the falling number of positions from PGY-2 programs (Figure 1). Solely focusing on the number of overall positions available, this trend maintains its nature, with the number of PGY-2 positions for anesthesiology dwindling over the years. However, during that same time, PGY-1 programs have nearly doubled the number of positions, more than compensating for the overall ability of those to match.

Match trends in anesthesiology residency

**Figure 2:**
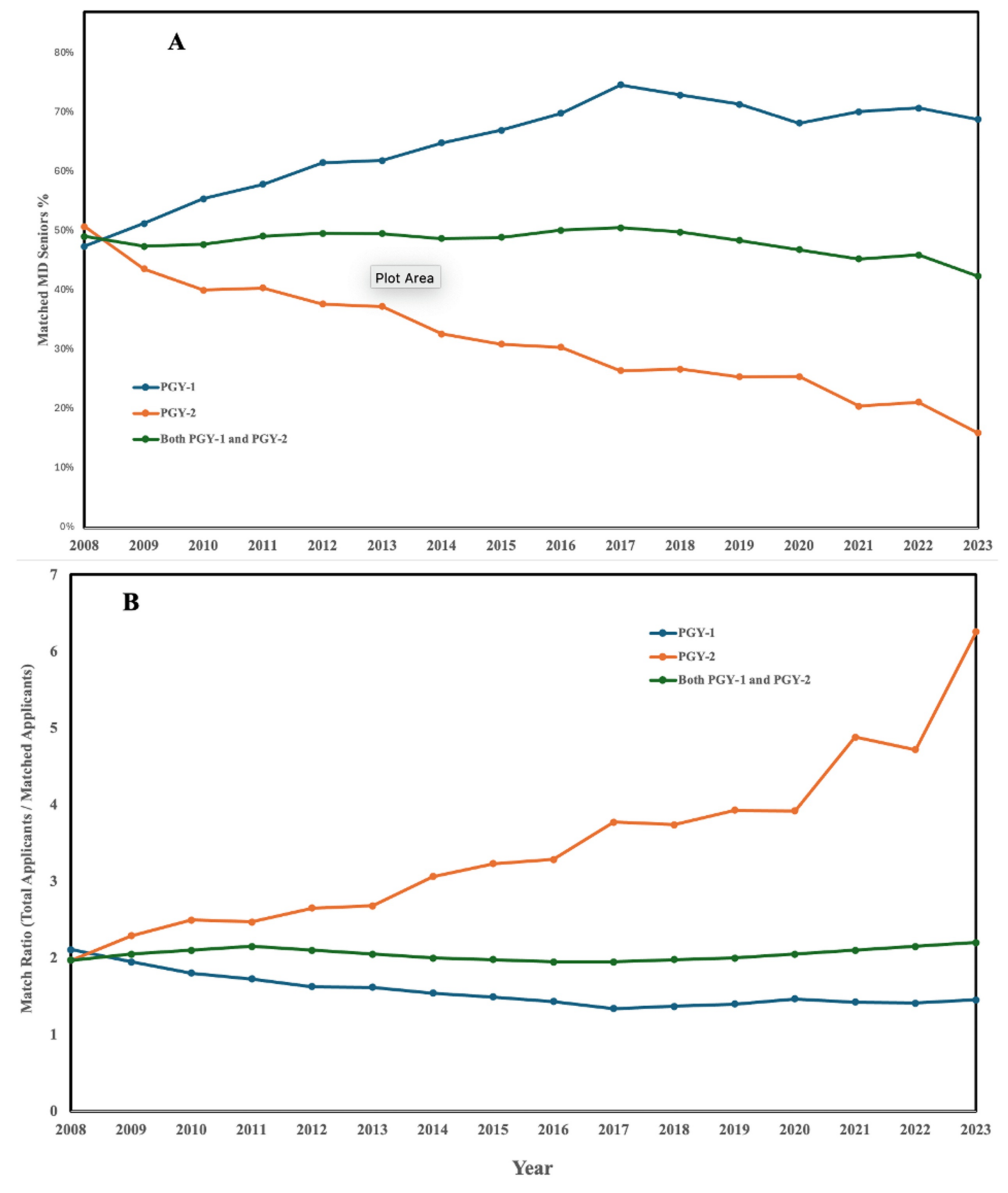
Match trends in anesthesiology residency Figure 2. Match trends in anesthesiology residency: A: PGY-1 match rates for medical doctor (MD) seniors have improved, with 1 in 1.5 applicants matching, B: while PGY-2 match ratios have worsened, with the total applicants per matched applicant increasing by over 300%, reflecting the growing competitiveness due to reduced PGY-2 opportunities.

Over the course of 2008 to 2023, the match rate of MD seniors has dramatically risen for PGY-1 programs, but the match rate for seniors to PGY-2 programs has had an equally dramatic fall. In order to better characterize the relative competitiveness of anesthesiology programs, we defined the “match ratio” as the total number of applicants to a program over the number of matched applicants for that program. Observing the change in match ratio over the course of the past 15 years paints a promising picture for MD seniors applying to PGY-1 programs, as their odds have somewhat improved over the years: for every 1.5 applicants, 1 gets accepted. However, PGY-2 match ratios paint a much bleaker picture, as the match ratio of total applicants per matched applicant has gone up by more than 300% in the same time frame. With this, it becomes apparent that the relative competitiveness of anesthesiology is at least in part largely driven by the diminishing PGY-2 program opportunities and the increased competitiveness to obtain one of those opportunities for MD seniors (Figure 2). 

Several factors, including mean contiguous ranks, mean distinct specialties, Alpha Omega Alpha (AOA) status, mean Step 2 score, mean research experiences, and mean abstracts, presentations, and publications, demonstrated statistically significant differences when comparing MAA to UAA and comparing MAA to AMA. Other factors, such as enrollment at a top 40 National Institute of Health medical school (p = 7.59 x 10⁻³), possession of a graduate degree (2.46 x 10⁻²), and mean Step 1 score (1.47 x 10⁻⁷) predicted a successful match only when comparing MAA to UAA. Possession of a PhD degree demonstrated variable results, with statistical significance in MAA to AMA (2.67 x 10⁻²) and multiple group (2.92 x 10⁻⁴) comparisons, but not a MAA to UAA comparison (2.7 x 10⁻¹) (Table 1).

**Table 1:** The comparison of different applicant groups to anesthesiology residency Note: NIH: National Institutes of Health, PhD: Doctor of Philosophy, AOA: Alpha Omega Alpha

	Multiple Group Comparison	Matched Applicants to Unmatched Applicant Comparison	Matched Applicants to All Matched Applicants Comparison
Mean Contiguous Ranks	p= 1.38 x 10^-4^	7.81 x 10^-3^	1.42 x 10^-2^
Mean Distinct Specialties	6.32 x 10^-9^	3.60 x 10^-4^	9.59 x 10^-5^
% from Top 40 NIH School	1.86 x 10^-4^	7.59 x 10^-3^	8.00 x 10^-2^
% PhD	2.67 x 10^-2^	2.7 x 10^-1^	2.92 x 10^-4^
% Graduate Degree	5.78 x 10^-2^	2.46 x 10^-2^	7.45 x 10^-2^
% AOA	7.54 x 10^-15^	8.90 x 10^-7^	3.14 x 10^-7^
Step 1 Mean	7.27 x 10^-7^	1.47 x 10^-7^	1.97 x 10^-1^
Step 2 Mean	2.48 x 10^-4^	1.01 x 10^-6^	9.35 x 10^-3^
Mean Research Experiences	7.29 x 10^-2^	5.66 x 10^-4^	3.32 x 10^-5^
Mean Abstracts, Presentations, and Publications	5.05 x 10^-2^	5.63 x 10^-3^	1.24 x 10^-4^

Trends of charting outcomes across the anesthesiology match over time

NRMP data showing specific charting outcome metrics were compared from 2007 to 2022. Initially, all 3 groups were compared to determine any significant differences between MAAs, UAA, and AMAs. Notably, mean abstracts, publications, and presentations over time (p = 5.05 x 10⁻²) and mean research experiences over time (7.29 x 10⁻²) did not showcase a significant difference across the 3 groups, and the trendlines over the years for these two metrics showcase that both metrics have increased consistently across all 3 groups over the years in a way that appears similar. While the percentage of applicants with graduate degrees (5.78 x 10-2) did not appear significant across the 3 groups, the percentage with a PhD (2.67 x 10-2) was significant, potentially due to the variable behavior of unmatched applicants over the years. Overall, it appears that many metrics are significant across multiple groups, but it is worth noting that MAAs are significantly different in behavior compared to UAAs (5.66 x 10-4) and AMAs (3.32 x 10-5) in both research-related outcomes when compared directly (Table 1).

## Discussion

**Figure 3:**
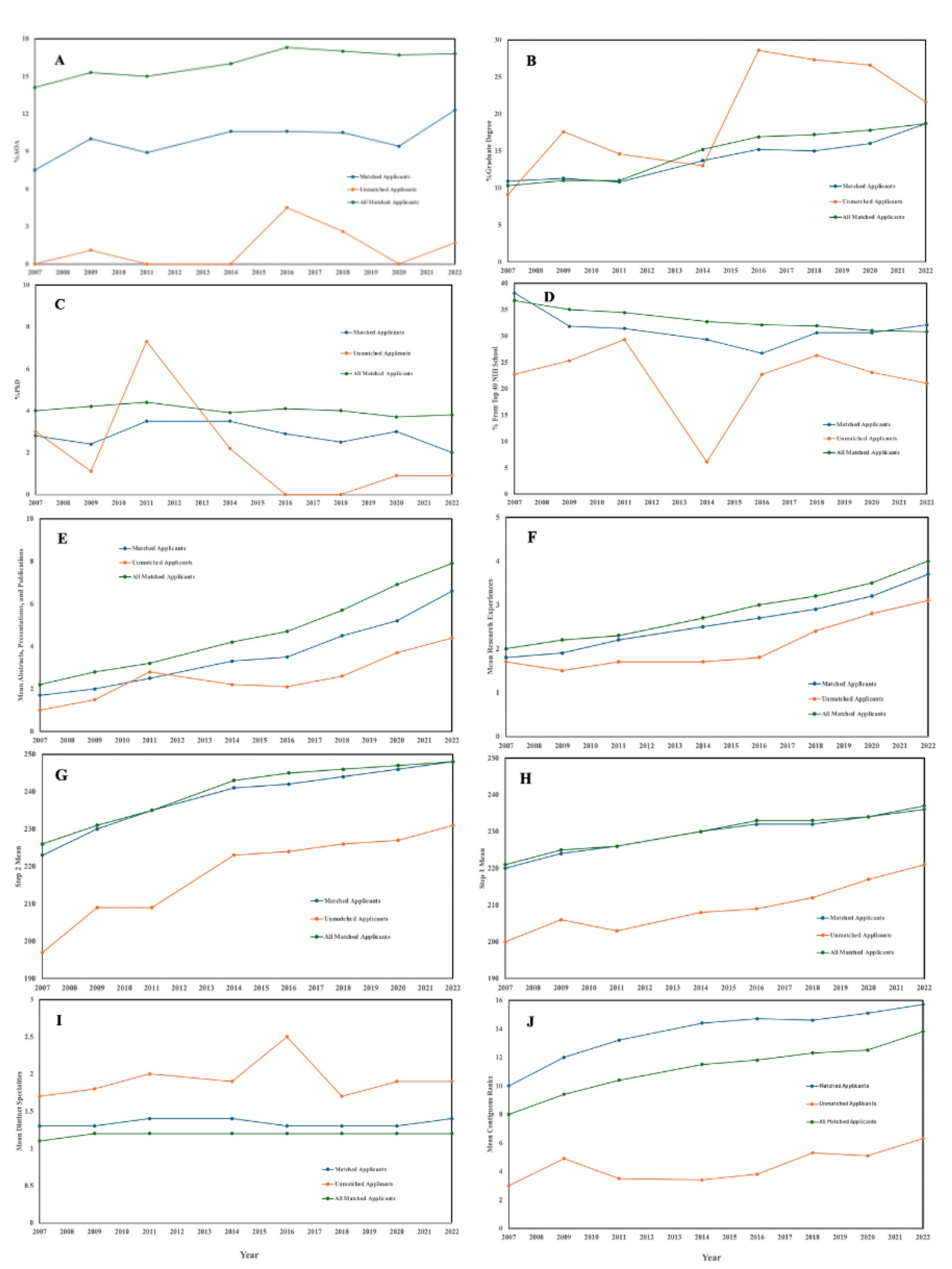
Comparison of multiple factors on applicant matching over time Figure 3. A: Trend showing comparison of AOA status among candidates over time, demonstrating consistently higher %AOA among matched compared to unmatched candidates. B: Comparison of % graduate degrees over time, showing an overall increase in the groups, with greater variability observed among unmatched candidates. C: Yearly variations in % PhD, with stabilized trends observed among matched applicants and greater fluctuations in unmatched candidates. D: Trend highlighting the variability of percentages from top 40 NIH schools over time among both unmatched and matched applicants. E: Progressive increase in the mean abstracts, presentations, and publications over time among all the groups. F: Steady increase in mean research experiences over time in both matched and unmatched applicants. G: Comparison of Step 2 mean over time, with higher scores observed in matched applicants vs. those unmatched. H: Greater Step 1 means observed over time for matched candidates compared to those unmatched. I: Comparison of distinct specialties over time, with a stable trend seen for matched applicants and a fluctuating pattern for those unmatched. J: Upward trajectory in contiguous ranks over time for matched candidates, and lower ranks observed for unmatched applicants.

The results from this study demonstrated that successfully matched applicants displayed increasing research and presentation experience in their applications. In the matched applicants' categories, the average research experience showed a 2-point increase. The unmatched applicant category also exhibits a slight increase in research experiences over time. The mean presentations and publications overtime in both the unmatched and matched applicants showed a significant but moderate upward trend as well throughout the years (Figures 3E, 3F). Other studies, such as one conducted by Matthews et al. that charted the outcomes of residency candidates with or without a history of research experiences, have reported similar findings [12]. This study showed that candidates from allopathic medical schools that had a research background were significantly more likely to have matched into anesthesia than their non-research-background counterparts [12]. Due to previous literature and our current findings, we believe that the importance of a candidate having general research experiences, as well as the number of such experiences, will increase compared to previous years.

This trend is also reflected in the average Step 1 and Step 2 scores. However, the average Step 2 scores showed a significantly larger increase in scores than its Step 1 counterparts, with Step 2 average scores showing an almost 30-point increase compared to Step 1 average scores that show an almost 20-point increase (Figures 3G, 3H). The trend can be assumed to carry through in the future for Step 1 scores due to its shift from a numeric scoring system to a pass/fail outcome in January of 2022. This change in the score reporting system stemmed from the USMLE’s concerns in multiple domains, including the perceived “overemphasis on Step 1 numeric scores as a screening tool in determining residency interviews and selection, Step 1’s outsized impact on the medical education curriculum, and the effect on student wellness” [13]. Thus, it is highly suggestive that licensing examination scores, specifically Step 2 scores, do play a key role in successful matching in anesthesia residencies. This is likely due to the elimination of numerical score-reporting for Step 1, which historically provided residency programs with an indication of a candidate’s competitiveness [14]. As a result, other numerical values in a candidate’s application, such as Step 2 scores, have become increasingly important. Our findings are also echoed in other studies that looked at the effect of replacing Step 1 with Step 2 scores in predicting student performance and matches [15]. In Berk et al., they found that although Step 2 scores showed a moderate correlation with Step 1 scores, using only Step 2 scores produced a different pool of applicants that would have been considered for review for a match than those produced by using both Step 2 scores and Step 1 scores [15]. As such, there are multiple variables aside from STEP 1 and STEP 2 scores that applicants must consider when aiming to match into their specialty of interest. This suggests the need to screen candidates in more comprehensive ways. Thus, we believe that while numerous other components of a student's application may be given more weight, this shift in emphasis will be most dramatically seen in Step 2 scores.

Next, we analyzed the impact of having a postgraduate degree on matching outcomes. The trend of successful match candidates who carried a graduate degree revealed a significant, slight increase throughout the years (Figures 3B, 3C). Interestingly, the trend of carrying specifically a PhD degree did not display the same significant increase in matched applicants versus their unmatched counterparts. This is consistent with previous literature indicating that students with an MD/PhD background did not outperform their MD-only counterparts in match outcomes for primary care specialties or more competitive specialties like anesthesia [16]. We believe that this may be because there are considerably fewer MD/PhD programs in the country than MD-only programs. In addition, we suggest that candidates who have PhDs prior to pursuing a medical degree are much rarer than students who obtain postgraduate degrees, such as a master's degree, during a gap year before enrolling in medical school. Thus, this would explain the discrepancy between the match outcome trends seen in candidates with graduate degrees versus candidates with PhD degrees. Other factors, such as Alpha Omega Alpha (AOA) membership in medical school and contiguous ranking in the residency applications, showed a significant increasing trend throughout the years in matched applicants versus unmatched applicants (Figures 3A, 3J). On the other hand, variables such as the top 40 NIH schools and the mean distinct specialties showed no real significant trend (Figures 3D, 3I). Overall, it is crucial to continue monitoring these broad trends for the anesthesiology match outcome to look for further variables that may affect the match rate for allopathic medical students after Step 1’s switch to a pass/fail system.

When considering the consequences of switching Step 1 to a pass/fail system, much attention has been drawn to the objective components of a residency application-namely, the increased emphasis on Step 2 CK scores. However, the remaining subjective counterparts should not be understated. Vinagre et al. found that once Step 1 is reported as pass/fail, as high as 80% of PDs ranked LORs among the top 10 factors for selecting applicants for interviews. The number of PDs who ranked LORs as a top 5 factor for selecting interviewees jumped from 28.9% to 42.3% when taking the new Step 1 grading system into consideration [8]. In a different study also surveying ACGME-accredited anesthesiology programs, Pierre et al. found that 53% of PD/APDs reported LORs as moderate to very important for granting an interview, but only 41% considered LORs important for generating a rank list [17]. This provides further evidence supporting the significant role of LORs in the interview determination process. LORs are one of the few narrative components in a candidate’s application that allow for PDs to assess a candidate’s fitness for their program specifically. A major strength of LORs is their ability for letter writers to incorporate personal anecdotes and detailed examples about the candidate in support of their strong qualifications. This narrative LOR allows for more personalization in writing style and elucidation of the precise relationship between the writer and the residency candidate. Interestingly, however, this type of LOR is not unanimously well-received among PDs. Pierre et al. report that 36% of PD survey respondents prefer standardized LORs (SLORs) to narrative LORs. The main justifications for their preference included more objective criteria, increased consistency among letters, and increased efficiency (a format that increases the ease of reading multiple letters) [17]. SLORs have received mixed results since their inception. In Pierre et al.’s study, the majority of anesthesiology PDs reported a preference for narrative LORs, a sentiment shared by surgery PD/APs, according to another study [17,18]. However, SLORs have been incorporated into other residency programs, including emergency medicine, otolaryngology, dermatology, and plastic surgery. Most notably, dissatisfaction with narrative LORs seems to stem from a lack of agreement on the definition of superlative adjectives used to describe residency candidates. A writer may describe a top-tier residency candidate as "excellent," but the reader may interpret this adjective as inferior to other descriptors such as “top [%] of the class” or “highest recommendation.” In fact, the same study found that 21% of anesthesiology PDs reported the term “strong” as a potential red flag. Overall, the lack of a standardized metric for interpreting verbiage leaves room for misinterpretation of the writer’s intention [17].

Eliminating a numerical step 1 score may inadvertently harm applicants from lesser-recognized medical schools, who may be reliant on the opportunity to distinguish themselves with a top percentile test score in order to receive an interview at a higher-ranked program. Consequently, these changes have resulted in the increasing prominence of a holistic review process that evaluates candidates, rather than hyper-focusing on any single entity of their application. With this approach to admissions, PDs not only assess a candidate's standardized test scores and medical school grades but also consider other aspects, including motivation for anesthesia, interpersonal skills and emotional intelligence, diversity, and evidence of grit in the face of hardships.

There are certain limitations in this study. First, although MD seniors are emphasized in the analysis of match rate for PGY-1 and PGY-2 positions, osteopathic applicants, international medical graduates, and reapplicants were not examined as independent cohorts, thus limiting subgroup-specific conclusions. Second, program-level variation was not evaluated. Anesthesiology residency programs were analyzed collectively, without accounting for differences in geographic location, academic reputation, resources, and selection patterns. This obscures the variability in competitiveness across the institutions. Finally, the interpretation of a favorable match outcome due to research productivity should be made cautiously. Research experiences and publications are self-reported in the NRMP data and reflect quantity instead of quality. The dataset does not differentiate important variables, such as first author versus co-author roles or high-impact publications compared to case reports. Overall, these limitations underscore the need for more granular, applicant-level, and program-specific analyses to refine the conclusions presented.

## Conclusions

In conclusion, our analysis of anesthesiology residency applications reveals several noteworthy trends. The transition to a pass/fail Step 1 has heightened the importance of other application components, such as Step 2 scores, research experience, and standardized letters of recommendation, which now play a greater role in a holistic evaluation of candidates. Despite the increase in match rates at PGY-1 programs, there is a concerning decline at PGY-2 programs, as evidenced by the match ratio of total applicants per matched applicant increasing by 300% over 15 years. In summary, our findings underscore the increasing competitiveness of matching into anesthesiology residency programs and the consequent need for applicants to adapt to the evolving trends in applicant qualifications and match outcomes.
